# The Association Between Breastfeeding and Atopic Dermatitis: An Epidemiological Analysis of Global and National Datasets

**DOI:** 10.7759/cureus.99441

**Published:** 2025-12-17

**Authors:** Xi Fu, Jieying Ou, Xinghan Wang, Yu Sun

**Affiliations:** 1 School of Public Health, Guangdong Pharmaceutical University, Guangzhou, CHN; 2 College of Life Sciences, South China Agricultural University, Guangzhou, CHN

**Keywords:** atopic dermatitis, breastfeeding, eczema, epidemiology, global burden of disease, nhanes, risk factors

## Abstract

Background and objective

The association between breastfeeding and the risk of atopic dermatitis (AD) in children is a subject of ongoing debate. While a broad consensus suggests a protective effect, several regional cohort studies have reported null or even risk-associated outcomes, creating clinical uncertainty. This study aimed to clarify this relationship by evaluating the association between breastfeeding practices and AD prevalence by using large-scale, population-level data.

Methods

We conducted a cross-sectional analysis using two independent, publicly available datasets. First, we used the Global Burden of Disease (GBD) 2019 study to analyze the correlation between AD prevalence in children aged one to four years and various risk factors across 204 countries using linear regression. Second, we analyzed data from the U.S. National Health and Nutrition Examination Survey (NHANES) 2005-2006 for 1,605 children aged one to six years, using logistic regression to assess the link between breastfeeding duration and doctor-diagnosed AD.

Results

The GBD analysis revealed that discontinuous breastfeeding was a highly significant risk factor for childhood AD (R² = 0.21, p<0.001) and remained a key independent predictor in the final multivariate model (p = 0.016). The NHANES analysis corroborated this finding, showing that children with AD had a significantly shorter median breastfeeding duration than healthy children (5.1 vs. 6.1 months, p = 0.03). Furthermore, each additional month of breastfeeding was associated with a 3% reduction in the odds of having AD (odds ratio (OR): 0.97, 95% confidence interval (CI): 0.95-0.999, p = 0.04).

Conclusions

Our findings from two large, independent, and geographically diverse datasets consistently demonstrate a protective association between breastfeeding and childhood AD. This evidence reinforces global public health recommendations and suggests that while local factors may modulate risk in specific cohorts, the overall benefit of breastfeeding for AD prevention is robust at the population level.

## Introduction

Atopic dermatitis (AD) is a common chronic inflammatory skin disease that primarily affects children, and its global prevalence has increased markedly over the past few decades [1,2]. The etiology of AD is multifactorial, involving a complex interplay of genetic predisposition, immune dysregulation, skin barrier dysfunction, and environmental exposures [1,3,4]. Early-life factors are considered critical in shaping immune development and subsequent AD risk, with infant feeding practices, particularly breastfeeding, being a central area of research [5]. A broad consensus supported by numerous meta-analyses suggests that exclusive breastfeeding for the first few months of life is a protective factor against the development of AD [5,6]. Breast milk contains a unique combination of nutrients, immunoglobulins, oligosaccharides, and beneficial microbes that are thought to promote a healthy gut microbiome and mature the infant's immune system, thereby reducing allergic sensitization [5].

However, the evidence is not entirely uniform, and the topic remains an area of active debate. The conflicting reports can be broadly differentiated into two types: large epidemiological studies reporting inconsistent associations, and mechanistic studies proposing biological pathways for a potential risk. On the epidemiological front, several large prospective cohort studies have reported null or even positive associations between breastfeeding and AD risk, challenging the protective consensus. For instance, a population-based UK cohort found no evidence that breastfeeding protected against eczema at any point from infancy through adolescence [7], while the large Danish National Birth Cohort found no overall protective effect and even reported an increased risk of AD with exclusive breastfeeding in children with no parental history of allergy [8].

Even more strikingly, a series of cohort studies suggested that prolonged breastfeeding might be a risk factor for atopic diseases. Notable examples include a longitudinal study in New Zealand, which linked breastfeeding for more than four weeks to an increased risk of atopy and asthma [9], and a German cohort where longer breastfeeding duration was a risk factor for atopic eczema [10]. Complementing these epidemiological observations, a potential biological basis for this risk was recently provided by the mechanistic study by Jiang et al., which linked high arachidonic acid in breast milk to gut dysbiosis and the onset of AD in a Chinese cohort [11].

A common thread among these conflicting reports is that they are often derived from single-center or regional cohorts. These studies, while invaluable for generating hypotheses, are susceptible to limitations such as smaller sample sizes and potential confounding from unmeasured local factors (e.g., specific dietary patterns, environmental exposures, or limited genetic diversity). This makes it difficult to generalize their findings to broader populations and highlights a critical gap: the need for analysis of large-scale, representative datasets to determine the overall, population-level association between breastfeeding and AD, averaged across these local variations. Therefore, our primary objective was to conduct an exploratory analysis to describe the association between breastfeeding patterns and AD prevalence using large-scale global and national datasets, including the Global Burden of Disease (GBD) project and the U.S. National Health and Nutrition Examination Survey (NHANES).

## Materials and methods

Global Burden of Disease (GBD) 2019 data analysis

We conducted an exploratory ecological study by using data from the GBD 2019 study [12]. The data were obtained from the GBD 2019 study, accessed via the official GBD results tool (https://vizhub.healthdata.org/gbd-results/). The risk factor "discontinued breastfeeding" was used as defined and modeled by the GBD project, which is based on the proportion of children not meeting the World Health Organization's recommendations for breastfeeding duration. The GBD 2019 dataset was selected to avoid potential confounding effects of the COVID-19 pandemic on healthcare access and environmental factors present in later datasets [12]. Data included the prevalence of AD for children aged one to four years and the summary exposure value for all 88 available risk factors across 204 countries.

Statistical analysis was performed using R (Version 4.1.0). First, a bivariate linear regression analysis was conducted to assess the individual association between each risk factor and AD prevalence. We used linear regression for this exploratory step to provide a straightforward measure of association (R²), acknowledging that assumptions of independence and homoscedasticity are likely violated in ecological data. Following a conventional strategy for exploratory model building, risk factors with a p-value <0.2 in the bivariate analysis were included as candidates in a multivariate linear regression model. A forward stepwise approach was used to build the final multivariate model to identify the most significant predictors of AD prevalence. A p-value of <0.05 was considered statistically significant.

National Health and Nutrition Examination Survey (NHANES) 2005-2006 data analysis

We further validated the association by using data from the NHANES 2005-2006 cohort [13], a nationally representative survey of the U.S. population. We selected a sub-sample of 1,605 children aged one to six years with available data on breastfeeding history and AD status. The presence of AD was determined by parental report of a doctor's diagnosis. Crucially, NHANES survey weights were not applied in this analysis; therefore, our findings are specific to this sample and are not nationally representative of the U.S. population.

Breastfeeding duration, recorded in months, was the primary exposure variable. The duration was compared between the AD group and the healthy control group by using the Mann-Whitney U test. A multivariate logistic regression model was used to calculate the odds ratio (OR) for the association between each additional month of breastfeeding and the prevalence of AD, with adjustment limited to the child's age and gender due to the availability of data in our working dataset. No formal plan for handling missing data was pre-specified. Statistical analyses were conducted using SPSS Statistics, Version 25.0 (IBM Corp., Armonk, NY), with a p-value <0.05 indicating significance.

## Results

Global association between risk factors and atopic dermatitis

In the bivariate analysis of GBD 2019 data, several risk factors were highly significantly associated with childhood AD prevalence (Table 1). Discontinuous breastfeeding showed the strongest positive association, with the model explaining 21% of the variance in AD prevalence (Coefficient = 0.0044, R² = 0.21, p<0.001). This was followed closely by secondhand smoke (Coefficient = 0.0017, R² = 0.20, p<0.001). Conversely, factors indicative of improved public health, such as access to handwashing facilities and safe water and sanitation, were all significantly and negatively associated with AD, indicating a protective effect (all p<0.001). Readers should interpret this exploratory bivariate analysis with caution due to the large number of comparisons made without statistical correction; primary conclusions are drawn from the more robust multivariate model (Table 2).

**Table 1:** Bivariate regression analysis of risk factors associated with childhood AD prevalence across 204 countries (GBD 2019) This table was created by the authors based on their analysis of the publicly available GBD 2019 dataset. Bivariate linear regression models were used to assess the individual association of each risk factor with AD prevalence. A p-value <0.05 was considered statistically significant AD: atopic dermatitis; GBD: Global Burden of Disease project; CI: confidence interval

Risk factors	Coefficients	Standard error	95% CI		Adjusted R^2^	P-value
Discontinued breastfeeding	0.0044	0.00079	0.0028	0.0060	0.21	0
Secondhand smoke	0.0017	0.00031	0.0011	0.0023	0.20	0
No access to a handwashing facility	-0.00076	0.00014	-0.0010	-0.0005	0.20	0
Unsafe water source	-0.0009	0.00017	-0.0013	-0.0006	0.19	0
Unsafe sanitation	-0.0008	0.00017	-0.0011	-0.0005	0.16	0
Household air pollution from solid fuels	-0.0012	0.00027	-0.0018	-0.0007	0.15	0
Child stunting	-0.0021	0.00053	-0.0032	-0.0011	0.12	0
Vitamin A deficiency	-0.0012	0.00032	-0.0019	-0.0006	0.11	0
Child wasting	-0.0094	0.00252	-0.0144	-0.0043	0.10	0
Child underweight	-0.0039	0.00110	-0.0060	-0.0017	0.09	0.001
Ambient particulate matter pollution	-0.0013	0.00044	-0.0022	-0.0004	0.06	0.004

**Table 2:** Multivariate regression analysis of risk factors for childhood AD prevalence (GBD 2019) This table was created by the authors based on their analysis of the publicly available GBD 2019 dataset. The final multivariate linear regression model was built using a forward stepwise approach to identify the strongest independent predictors of AD prevalence (overall model R² = 0.29, p<0.001). A p-value <0.05 was considered statistically significant AD: atopic dermatitis; GBD: Global Burden of Disease project; CI: confidence interval

Risk factors	Unstandardized coefficients	Standard error	Standardized coefficients	P-value	P-value (model)	Adjusted R^2^
Secondhand smoke	0.001	0.00034	0.334	0	0	0.29
Discontinued breastfeeding	0.002	0.00093	0.238	0.016		
Ambient particulate matter pollution	-0.001	0.00042	-0.176	0.041		

When these factors were entered into a final multivariate model to identify the strongest independent predictors, three remained statistically significant (overall model R² = 0.29, p<0.001; Table 2). Secondhand smoke emerged as the most significant risk factor (standardized β = 0.334, p<0.001), followed closely by discontinuous breastfeeding (standardized β = 0.238, p = 0.016). Ambient particulate matter pollution was also retained in the model (standardized β = -0.176, p = 0.041). These findings highlight that, at a global level, a lack of continuous breastfeeding is one of the primary modifiable risk factors for a higher prevalence of AD in children.

Association between breastfeeding duration and atopic dermatitis in a U.S. cohort

The analysis of 1,605 children from the NHANES 2005-2006 dataset corroborated the GBD findings. Of the participants, 267 (16.5%) reported a doctor's diagnosis of AD. As shown in Table 3, the healthy group had a significantly longer median breastfeeding duration than the AD group (6.1 months vs. 5.1 months, p = 0.03). The adjusted logistic regression analysis revealed that each additional month of breastfeeding was associated with a 3% reduction in the odds of having AD (OR: 0.97, 95% CI: 0.95-0.999, p = 0.04). Also, low birth weight, exposure to environmental tobacco smoke, and maternal smoking during pregnancy were not significantly different between the AD and healthy groups (p>0.05; Table 3), suggesting that these were not major confounders in this cohort.

**Table 3:** Characteristics of the NHANES 2005-2006 study cohort by AD status (n = 1,605) This table was created by the authors based on their analysis of the publicly available NHANES 2005-2006 dataset. Data are presented as median (IQR), mean ± SD, or n (%). P-values were calculated using the Mann-Whitney U test for breastfeeding duration, the independent samples t-test for age, and the chi-squared test for categorical variables. A p-value <0.05 was considered statistically significant NHANES: U.S. National Health and Nutrition Examination Survey; AD: atopic dermatitis; IQR: interquartile range; SD: standard deviation; ETS: environmental tobacco smoke

Variables	Overall cohort	Health group (n = 1,338)	AD group (n = 267)	Test statistic	P-value
Breastfeeding duration, months, median (Q1-Q3)	6.0 (2.0-10.7)	6.1 (2.0-11.1)	5.1 (2.0-8.1)	Z = 2.17	0.03
Male, n (%)	799 (49.8)	656 (49.0)	143 (53.7)	χ² = 2.00	0.158
Age, years, mean ± SD	3.16 ± 0.04	3.16 ± 0.05	3.15 ± 0.10	t = -0.12	0.904
Low birth weight (<2.5 kg), n (%)	220 (13.7)	177 (13.2)	43 (16.2)	χ² = 1.62	0.203
High birth weight (>4.0 kg), n (%)	23 (1.4)	19 (1.4)	3 (1.1)	χ² = 0.10	0.748
Exposure to ETS, n (%)	218 (13.6)	183 (13.7)	35 (13.2)	χ² = 0.05	0.819
Maternal smoking during pregnancy, n (%)	202 (12.6)	169 (12.6)	34 (12.8)	χ² = 0.01	0.906

When these factors were entered into a final multivariate model to identify the strongest independent predictors, three remained statistically significant (overall model R² = 0.29, p<0.001). Secondhand smoke emerged as the most significant risk factor (standardized β = 0.334, p<0.001), followed closely by discontinuous breastfeeding (standardized β = 0.238, p = 0.016). Ambient particulate matter pollution was also retained in the model (Standardized β = -0.176, p = 0.041). These findings highlight that, at a global level, a lack of continuous breastfeeding is one of the primary modifiable risk factors for a higher prevalence of AD in children.

## Discussion

Our analysis of two large, independent epidemiological datasets consistently showed that breastfeeding is associated with a reduced risk of AD in children. This finding aligns with the predominant body of literature and reinforces current public health recommendations. However, it contrasts with localized studies that have reported null or even risk-associated effects. A critical discussion is needed to reconcile these apparently contradictory results. A potential explanation for this discrepancy lies in the scale of observation and the influence of unmeasured local factors. Datasets like GBD and NHANES are powerful because their vast size and diverse populations reveal the net average effect of an exposure. They provide a robust, generalizable signal that is essential for forming broad public health policy.

In contrast, localized cohort studies, while invaluable for mechanistic insights, are highly sensitive to regional variables. Factors such as maternal diet, exposure to environmental pollutants, local microbial environments, and population-specific genetic predispositions can create conditions where breastfeeding's benefits are negated or even reversed in a specific subgroup [1,11]. For example, maternal dietary intake of fatty acids is known to directly influence the concentration of arachidonic acid and other polyunsaturated fatty acids (PUFAs) in breast milk [14], making the findings of Jiang et al. highly dependent on the dietary patterns of their specific cohort. Furthermore, population-specific genetic predispositions, such as common mutations in the filaggrin gene (FLG), which are a primary risk factor for AD, can dramatically increase susceptibility and may interact with early-life exposures in ways that vary between populations [15]. The findings from these regional studies may therefore be valid observations within their specific cohorts, but our results suggest this is not the globally dominant trend.

For these reasons, the results from GBD and NHANES should be given significant weight when formulating universal health recommendations. While mechanistic studies can identify potential biological pathways of harm, large-scale epidemiological data tell us how these factors play out in the real world across millions of individuals. Our findings suggest that, on balance, the multitude of protective components in breast milk overwhelmingly outweighs any potential risks for the majority of the global population. It is important to acknowledge that the observed effect size in the NHANES data is modest on a per-month basis (OR: 0.97); however, the clinical significance of this small effect may become substantial when accumulated over the recommended 6-12 months of breastfeeding.

The primary contribution of this study is to serve as a preliminary, hypothesis-generating exploration that provides an essential epidemiological anchor for the current debate. By analyzing large-scale population data, our findings provide a broad, generalizable counterpoint to the valuable but potentially context-specific results from smaller regional or mechanistic cohorts. It highlights the need for more rigorous research while suggesting that, at a broader level, the net association of breastfeeding appears protective.

The main strength of this study is its use of two large, publicly available datasets. However, the limitations are substantial and severely constrain the conclusions that can be drawn. First and most critically, the GBD analysis is ecological and subject to the ecological fallacy; associations observed at the country level cannot be assumed to apply to individuals. Second, our NHANES analysis did not incorporate the complex survey design weights, meaning the results are not nationally representative and may be biased. Third, the cross-sectional nature of the NHANES data makes it impossible to determine temporality, and the results are highly vulnerable to reverse causation. Fourth, the NHANES model is critically under-adjusted, lacking data on major known confounders such as parental atopy and socioeconomic status. Finally, the NHANES analysis found a statistically significant but very modest association (OR: 0.97), with a CI close to the null. This result is likely not robust and is highly susceptible to the aforementioned residual confounding.

Our findings, together with the study's significant limitations, underscore the need for more definitive research. The ideal next step would be to conduct large, prospective, multi-center longitudinal birth cohort studies. Such studies should be designed to collect detailed, repeated data on infant-feeding practices (including exclusivity), clinically diagnosed AD outcomes, and a comprehensive set of potential confounders. Crucially, they should also incorporate the collection of biological samples, such as breast milk to analyze its composition (e.g., PUFA ratios) and infant stool samples to track microbiome development. Integrating large-scale epidemiology with detailed biological and environmental data in this way is the only path to fully unraveling the complex relationship between breastfeeding and AD.

## Conclusions

In this exploratory analysis of two large observational datasets, we found a consistent statistical association between breastfeeding and a lower prevalence of childhood AD. However, due to major methodological limitations - including the potential for ecological fallacy, the lack of survey weighting in the NHANES analysis, extensive unmeasured confounding, and the inability to rule out reverse causation - these findings cannot establish a protective or causal relationship. Our study highlights the complexity of this research question and underscores the need for large, prospective longitudinal studies with detailed data on confounders to truly clarify the role of breastfeeding in AD.
